# The greenhouse gas budget for China's terrestrial ecosystems

**DOI:** 10.1093/nsr/nwad274

**Published:** 2023-10-27

**Authors:** Xuhui Wang, Yuanyi Gao, Kai Wang, Yuxing Sang, Yilong Wang, Yuzhong Zhang, Songbai Hong, Yao Zhang, Wenping Yuan

**Affiliations:** Institute of Carbon Neutrality, Sino-French Institute for Earth System Science, College of Urban and Environmental Sciences, Peking University, China; Institute of Carbon Neutrality, Sino-French Institute for Earth System Science, College of Urban and Environmental Sciences, Peking University, China; Institute of Carbon Neutrality, Sino-French Institute for Earth System Science, College of Urban and Environmental Sciences, Peking University, China; Institute of Carbon Neutrality, Sino-French Institute for Earth System Science, College of Urban and Environmental Sciences, Peking University, China; State Key Laboratory of Tibetan Plateau Earth System, Resources and Environment (TPESRE), Institute of Tibetan Plateau Research, Chinese Academy of Sciences, China; Key Laboratory of Coastal Environment and Resources of Zhejiang Province, School of Engineering, Westlake University, China; Institute of Advanced Technology, Westlake Institute for Advanced Study, China; Institute of Carbon Neutrality, Sino-French Institute for Earth System Science, College of Urban and Environmental Sciences, Peking University, China; Institute of Carbon Neutrality, Sino-French Institute for Earth System Science, College of Urban and Environmental Sciences, Peking University, China; School of Atmospheric Sciences, Sun Yat-sen University, China

## Abstract

The first greenhouse gas (GHG) budget accounting over China shows that China's land ecosystems is close to GHG neutral, in contrast to the net GHG source of global land ecosystems.

The increased atmospheric concentrations of greenhouse gases (GHGs, including CO_2_, CH_4_ and N_2_O) are unequivocally the major driving forces of climate warming [1]. These GHGs originate not only from the use of fossil fuels, but also from disturbances to and management of terrestrial ecosystems. Recent evidence suggests that terrestrial ecosystems, including various land ecosystems and inland water bodies, have become a net source of GHGs [2]. Reducing these ecosystem GHG emissions is therefore of immense importance for climate change mitigation [3]. Indeed, ecosystem GHG emission reduction is also a key component of the recently emerging natural climate solutions (NCS), which have attracted particular interest in China [4]. However, the lack of a comprehensive understanding of China's GHG budget has hindered proper assessment and large-scale application of NCS, impeding the nation's ambition to achieve carbon and eventually climate neutrality. To fill this crucial knowledge gap, here we provide a comprehensive GHG budget of China during the 2000s and 2010s with the dual constraint approach from both the bottom-up estimates (based on ground inventories and biogeochemical models) and the top-down estimates (based on atmospheric inversions).

We conducted a thorough GHG budget assessment over China, encompassing ∼40 budget terms. Details of the assessment framework can be found in [5], which aligns consistently with the methodology and terms used by the Global Carbon Project [6]. Specifically, for the bottom-up estimate, we gathered data from 31 ground inventories (11 for CO_2_, 10 for CH_4_ and 10 for N_2_O, respectively) and 48 biogeochemical models (18 for CO_2_, 23 for CH_4_ and 7 for N_2_O, respectively). Meanwhile, the top-down estimate involved 17 atmospheric inversions (10 for CO_2_, 3 for CH_4_ and 4 for N_2_O, respectively). By utilizing this multi-model and multi-data-source approach, we are able to provide a comprehensive assessment for all GHG fluxes of terrestrial ecosystems in China, while substantially minimizing the potential risk of a single biased model/flux source on the overall GHG budget. To assess the overall greenhouse effect, our budget combines CH_4_ and N_2_O with CO_2_, based on greenhouse warming potential (GWP, in CO_2_ equivalent) at the 100-year horizon [1]. GWP measures the cumulative impacts that the emission of 1 g of greenhouse gas could have on the planetary energy budget relative to 1 g of reference CO_2_, which mainly depends on the molecular structure and the lifetime in the atmosphere [2] (see also Supplementary Methods).

According to our best estimate, China's terrestrial ecosystems act as a small GHG sink (–29.0 ± 207.2 Tg CO_2_-eq yr^−1^ with the bottom-up estimate and –75.3 ± 496.8 Tg CO_2_-eq yr^−1^ with the top-down estimate; Fig. 1). By contrast, global terrestrial ecosystems in general release more GHG into the atmosphere than they absorb [2]. When differentiating terrestrial ecosystems into natural ecosystems and agricultural ecosystems using the bottom-up estimate, we find a much larger net sink of GHGs in China's natural ecosystems (–838.4 ± 167.0 Tg CO_2_-eq yr^−1^; Supplementary Table S1), which, however, is largely cancelled out by GHG emissions from agricultural ecosystems. Hence, reducing GHG emissions from agricultural ecosystems should be the priority for increasing the overall net GHG sink of terrestrial ecosystems in China. Furthermore, the small net sink of GHGs is also a result of a larger net CO_2_ sink, offset by net sources of CH_4_ and N_2_O (Supplementary Table S2).

**Figure 1.**
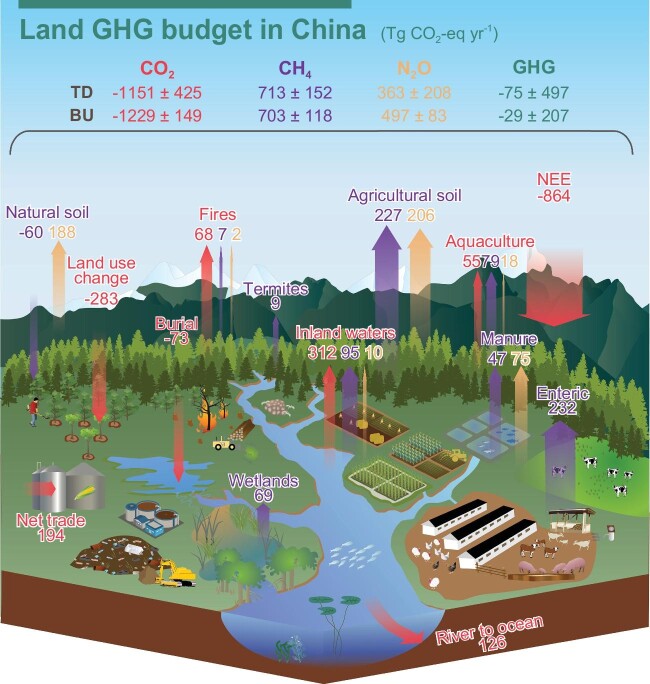
The greenhouse gas (GHG) budget for China's terrestrial ecosystems during the 2000s and 2010s.

China's terrestrial ecosystems are a significant net CO_2_ sink (–1151.0 ± 425.1 Tg CO_2_ yr^−1^ with the top-down estimate and –1229.2 ± 149.1 Tg CO_2_ yr^−1^ with the bottom-up estimate). It is also noteworthy that, with lateral flux adjustments, the top-down and bottom-up estimates of the CO_2_ budget show only a 6% difference, demonstrating recent methodological progress [7]. This convergence instills confidence in the accuracy of forthcoming CO_2_ stocktake assessments under the United Nations Framework Convention for Climate Change. Furthermore, China's land ecosystem CO_2_ sink contributes ∼20% to the contemporary global land CO_2_ sink despite occupying only 7% of the global land area [8]. More than half of China's terrestrial ecosystem CO_2_ sink is attributed to forest ecosystems (Supplementary Table S2), primarily due to large-scale afforestation and reforestation efforts.

Regarding CH_4_, terrestrial ecosystems in China are a net source of methane emissions (26.1 ± 4.4 Tg CH_4_ yr^−1^ with the bottom-up estimate and 26.4 ± 5.6 Tg CH_4_ yr^−1^ with the top-down estimate). The primary contributors to these CH_4_ emissions are enteric fermentation and paddy rice cultivation, accounting for ∼60% of the net CH_4_ source (Fig. 1 and Supplementary Table S2). Only non-saturated natural soil acts as a sink for CH_4_ at –2.2 ± 0.2 Tg CH_4_ yr^−1^.

Similarly, China's terrestrial ecosystems are also a net source of N_2_O (1.8 ± 0.3 Tg N_2_O yr^−1^ with the bottom-up estimate and 1.3 ± 0.8 Tg N_2_O yr^−1^ with the top-down estimate). N_2_O also exhibits the largest relative difference between the top-down and bottom-up estimates (∼25%) among the three GHGs, probably resulting from the sparsity of accessible atmospheric N_2_O observations for the inversion models [9]. The sectorial analysis shows that cropland N_2_O emissions from nitrogen fertilizer application are the single largest N_2_O source at 0.8 ± 0.3 Tg N_2_O yr^−1^ (Fig. 1 and Supplementary Table S2).

In summary, we have presented the first comprehensive GHG budget for terrestrial ecosystems in China. The integration of both bottom-up and top-down approaches, together with lateral adjustments, allows us to more confidently generate best estimates of the GHG budget (e.g. [10]). Although the existing wide array of accounting methods has made possible a comprehensive picture of the diverse contribution of terrestrial ecosystems to the GHG budget, each method offers its own benefits, as well as challenges and uncertainties. For example, although satellite GHG measurements have grown quickly in recent years, current atmospheric inversions still lack sufficient information from atmospheric observations over China, leading to the high sensitivity of posterior estimates to prior information (e.g. [11]). Such persistent challenges call for efforts to speed up the establishment of a measurable, reportable and verifiable system for GHG accounting.

Our results also imply the crucial importance and careful consideration needed for curbing GHG emissions. Because agricultural CH_4_ and N_2_O emissions offset >90% of the land CO_2_ sink, curbing agricultural GHG emissions will probably attract increasing attention in the agenda to mitigate climate change. A successful mitigation strategy will have to rely on sufficiently scrutinized solutions, which should address GHG emissions reduction without endangering the food supply for China's >1 billion people and provide co-benefits for the environment (e.g. [12]). This represents a huge sustainability challenge that urgently requires further studies.

## Supplementary Material

nwad274_Supplemental_File
