# Supplementary material for: The greenhouse gas budget for China's terrestrial ecosystems
Source: Natl Sci Rev. 2023 Oct 27;10(12):nwad274. doi: 10.1093/nsr/nwad274 (PMC10689210; doi:10.1093/nsr/nwad274)
Supplement: nwad274_Supplemental_File [file nwad274_supplemental_file.docx]

***Supplementary Information for***

**The greenhouse gas budget of terrestrial ecosystems in China**

Xuhui Wang^1^, Yuanyi Gao^1^, Kai Wang^1^, Yuxing Sang^1^, Yilong Wang^2^, Yuzhong Zhang^3,4^, Songbai Hong^1^, Yao Zhang^1^, Wenping Yuan^5^

*^1^Institute of Carbon Neutrality, Sino-French Institute for Earth System Science, College of Urban and Environmental Sciences, Peking University, Beijing, China; ^2^* *State Key Laboratory of Tibetan Plateau Earth System, Resources and Environment (TPESRE), Institute of Tibetan Plateau Research, Chinese Academy of Sciences, Beijing, China; ^3^Key Laboratory of Coastal Environment and Resources of Zhejiang Province, School of Engineering, Westlake University, Hangzhou, China; ^4^Institute of Advanced Technology, Westlake Institute for Advanced Study, Hangzhou, China; ^5^School of Atmospheric Sciences, SUN YAT-SEN University, Guangdong, China.*

***Supporting Text***

[1. Data 3](#_Toc140321591)

[1.1 CO_2_ datasets 3](#_Toc140321592)

[1.1.1 Top-down 3](#_Toc140321593)

[1.1.2 Bottom-up 4](#_Toc140321594)

[1.2 CH_4_ datasets 8](#_Toc140321595)

[1.2.1 Top-down 8](#_Toc140321596)

[1.2.2 Bottom-up 9](#_Toc140321597)

[1.3 N_2_O datasets 10](#_Toc140321598)

[1.3.1 Top-down 10](#_Toc140321599)

[1.3.2 Bottom-up 11](#_Toc140321600)

[2. Methods 12](#_Toc140321601)

[2.1 Reconcile top-down and bottom-up estimates 12](#_Toc140321602)

[2.1.1 CO_2_ 12](#_Toc140321603)

[2.1.2 CH_4_ 13](#_Toc140321604)

[2.1.3 N_2_O 14](#_Toc140321605)

[2.2 Integration of GHG budget 14](#_Toc140321606)

[2.3 Uncertainty estimate 15](#_Toc140321607)

[Table S1. Terrestrial GHG budget based on GWP100 and GWP20 metrics. 16](#_Toc140321608)

[Table S2. The GHG budget in China since 2000 17](#_Toc140321609)

[*Supporting References* 19](#_Toc140321610)

Following Wang et al.^1^ , we present here the first version of the synthesis dedicated to the national land greenhouse gas budget from CO_2_, CH_4_ and N_2_O, integrating results of top-down studies (satellite or in situ atmospheric observations within an atmospheric inverse-modelling framework) and bottom-up estimates (including multi-source inventories of agricultural and natural sinks and emissions, biogeochemical models for estimating land surface emissions, and data-driven extrapolations).

# 1. Data

## 1.1 CO_2_ datasets

### 1.1.1 Top-down

For the top-down constraints on the CO_2_ fluxes of China, we obtain estimates from 10 atmospheric inversion results, 3 of which come from the state-of-the-art inversion systems using regional background CO_2_ mole fraction measurements (GB) data or China-focused version of carbon assimilation system.

Jiang et al.^2^ estimated the CO_2_ fluxes in China during 2006-2009 using two well-established inversion systems, a nested Bayesian inversion (BI) system and the Carbon Tracker-China (CTC) system. Their inversion results were constrained with 3 sites from China Meteorological Administration (CMA)’s measurements (LFS, SDZ, and LAN) and the aircraft CO_2_ measurements from the Comprehensive Observation Network for Trace gases by Air Liner project (CONTRAIL) beyond the global background CO_2_ stations. When both CMA and CONTRAIL data are assimilated, the mean inverted CO_2_ sink in China is -1650 ± 917 Tg CO_2_ yr^-1^, which is a higher than the estimates without new CO_2_ data.

Chen et al.^3^ designed five inversions including different CMA sites (from 3 sites, including LFS, SDZ, and LAN to 6 sites, including LFS, SDX, LAN, WLG, JS, and AKDL) to investigate the impacts of additional atmospheric CO_2_ observations on estimate of the carbon sink in China. The more atmospheric CO_2_ observations involved, the greater the averaged magnitude of inverted carbon sinks they got, from -917 Tg CO_2_ yr^-1^ (3 CTC sites) to -1357 Tg CO_2_ yr^-1^ (6 CTC sites) and to -990 Tg CO_2_ yr^-1^ with additional GOSAT ACOS XCO_2_ data (CTC-7). Their magnitude of estimated flux by the CTC’s ensemble is smaller than the uncertainty (Fig 1b in Chen et al.^3^), suggesting that the inverted surface fluxes in most regions of China were still poorly constrained by sparse observation stations over China.

Recently, Wang et al.^4^ evaluated the representativeness error of the regional CMA site (SL) with complex orography by performing a factorial analysis using the inversion system from CAMSv19. The simulation results showed high sensitivity to the SL site, suggesting the biases of representing Shangri-La site observations could have led to the extremely large inverse estimate. Their estimate (-1430 Tg CO_2_ yr^-1^) eliminating the controversial site during 2010-2016 is used in this study.

### 1.1.2 Bottom-up

The magnitude of regional land CO_2_ budget can be quantified by the annual carbon stock changes (△C)^5^ or the critical terrestrial ecosystem process simulations^6^.

We have reconstructed carbon stock changes of vegetation and soil in China since 2000 using inventory-satellite-model based estimates, the carbon stock changes areas calculated as the sum of fluxes from above-ground and below-ground carbon storage changes in forests (${\Delta C}_{forest}$), grasslands (${\Delta C}_{grassland}$), shrublands (${\Delta C}_{shrubland}$) croplands (${\Delta C}_{cropland}$), other natural carbon stocks (${\Delta C}_{other}$, including wetlands and urban ecosystems), carbon burials in sediments (${\Delta C}_{burial}$) and crop and wood products (${\Delta C}_{product}$). Results for China extracted from global-scale datasets FAOSTAT and OSCAR refer to Wang et al.^1^. Country-specific estimates have also been integrated, the detailed national datasets we have used are as follows.

#### △C

The National Communication on Climate Change of The People’s Republic of China (NCCC)^7, 8^ compiled the inventories of main GHGs (e.g. CO_2_, CH_4_, N_2_O, etc.), covering five main areas (including energy activities, industrial production processes, agricultural activities, land cover and land use change, and waste treatment). NCCC adopted the high-level methodology provided by IPCC guidelines based on the activity data from national statistical yearbook as well as constantly updated national emission factors. Using data from the 6th (1999–2003) to 9^th^ (2014–2018) continuous national forest resource inventories, the NCCC integrated activity level data of the provinces, as well as emission factors of the national forest inventory and soil carbon emission factors of agricultural land to estimate country-level ${\Delta C}_{forest}$, ${\Delta C}_{cropland}$, ${\Delta C}_{grassland}$, ${\Delta C}_{other}$, and ${\Delta C}_{product}$ for 2000, 2005, 2010, 2012 and 2014, the mean result for these years have been used in our synthesis.

Piao et al.^9^ analyzed the terrestrial carbon balance of China during the 1980s and 1990s using biomass and soil carbo inventories extrapolated by satellite greenness measurements, ecosystem models and atmospheric inversions. Although the time span of the study is beyond ours, the statistical functions they established to estimate biomass and soil carbon are still conductive. For the biomass parts of ${\Delta C}_{grassland}$ and ${\Delta C}_{shrubland}$, based on the relationship between NDVI and aboveground biomass, they reported the carbon stock changes as -80 ± 37 Tg CO_2_ yr^-1^ and -26 ± 9 Tg CO_2_ yr-1. For the soil carbon for natural ecosystems, similar to the estimation of biomass carbon stock, we used their statistical function integrating climate data (temperature and precipitation), NDVI data or biomass data, and ground-based soil inventory data. The subtotal of soil carbon stock changes was -276 ± 92 Tg CO_2_ yr^-1^.

Pan et al.^10^ estimated forest biomass C stock and its change during the 1990s and 2000s for China. Using biomass expansion factors for each forest type and China’s forest inventory data since 1990s, they established a robust linear relationship for the forest area and timber volume at the provincial level, then calculated carbon in soil based on ratios of soil C to vegetation biomass. They estimated ${\Delta C}_{forest}$ was -640 ± 165 Tg CO_2_ yr^-1^ during 2000-2007. For the carbon flux in harvested wood products, they derived a conversion factor from the countries that report the ratio of C in harvested wood to the quantity of harvested roundwood according to FAO reports, and the ${\Delta C}_{product}$ was reported as -27 Tg CO_2_ yr^-1^.

Jiang et al.^2^ reconstructed carbon stock changes of vegetation and soil in China during 2000s based on previous estimates concerning forests biomass carbon stock accumulation, and the 6th (1999–2003) and 7th (2004–2008) national forest inventories. They reported the bottom-up carbon exchange estimate as -477 ± 139 Tg CO_2_ yr^-1^.

For ${\Delta C}_{burial}$ inland aquatic ecosystems, they estimated carbon buried in the sediments of lakes based on two previous studies of organic carbon burial rates in six lakes in the middle and lower reaches of the Yangtze River Basin during 2000s, for the reservoirs, they assumed that the carbon burial rate in Chinese reservoirs was about two times of the global mean rate. The subtotal estimate of ${\Delta C}_{burial}$ during 2000s is -73 ± 37 Tg CO_2_ yr^-1^.

Wang et al.^4^ updated the carbon burial flux in China during 2010-2016 based on a generated OC burial dataset^11^ in lakes and reservoirs from a meta-analysis of global modern (last ~150 years) whole-basin OC burial literature data.

Fang et al.^12^ summarized the major findings relating to the magnitudes and changes of carbon pools in each ecosystem carbon sector from 2001 to 2010, mainly based on the Strategic Priority Project of Carbon Budget supported by the Chinese Government. A total of 17,090 constant field plots were sampled in the project, of which 7,800 plots were from forests, 1,200 plots from shrublands, 4,030 plots from grasslands, and 4,060 soil sites from croplands. A total of -737 Tg CO_2_ were collectively sequestrated by ecosystems during 2000s from their estimate, in which forests contributed the most (80%), followed by cropland (12%) and shrubland (8%). The results have been gathered into our integration work.

In addition to inventory-based approach, some satellite-based estimates are included in our synthsis. Xu et al.^13^ developed spatially explicit estimates of carbon stock changes of live biomass from 2000-2019 combining a large number of ground inventory plots (> 100,000), airborne and satellite data as a consistent set of measurements, with machine learning models. We extract spatio-temporal results for China from their dataset.

Chang et al.^14^ estimated live woody aboveground biomass carbon (AGC) dynamics over China between 2013 and 2019 using 3 microwave (L- and X-band vegetation optical depth [VOD]) and 3 optical (normalized difference vegetation index, leaf area index, and tree cover) remote-sensing vegetation products. Their results of the AGC estimation model showed that carbon uptake by the forests in China was about -612 Tg CO_2_ yr^-1^ from 2013 to 2019. We complete the below-ground biomass (BGC) for this estimate by referring to the isometric growth rate relationship between AGC and BGC of forests in China based on the observation-based result from Fang et al.^15^.

#### Lateral transport

For the carbon contained in net imports of forest and crop products from outside China, we refer to two estimates^2, 4^ based on the import and export data of crop and wood products from the FAO statistical databases.

For the river export of carbon delivered to ocean or across region boundaries ($F_{export}$), two national estimates as follows have been contained in our integration.

Jiang et al.^2^ evaluated DOC, DIC, and POC based on the observations from nine Chinese exorheic rivers during 2006-2009 and empirical formula from previous studies.

Yan et al.^16^ proposed the first DOC modeling effort applying a random forest model with a harmonized database of riverine in-situ measurements. Their model is capable of reproducing well the magnitude of riverine DOC flux, with a much wider spatial distribution over China especially the Southwest Rivers Basin compared to previous studies. Their result showed that over the period 2001–2015 the average DOC flux was 4.0  ± 1.0 Tg C-DOC yr^-1^.

#### Biogeochemical model estimates of NBP

In this study, the TRENDY v9 dataset based on 18 Dynamic Global Vegetation Models (DGVMs)^6^ have been run to estimate the NBP in China. Spatially gridded NBP were obtained with simulation 2 (S2) from up to 2019.

#### Other natural sectors

As we have used the NBP estimates from TRENDY models forced by constant land cover, the net CO_2_ flux from land cover, land use change, and forestry ($F_{luc}$) should be estimated separately. In addition to global estimates (BLUE, OSCAR, H&N2017, TRENDY simulations) which have been mentioned in Wang et al.^1^, two national estimate in China have been integrated as follows.

Yu et al.^17^ reconstructed a new gridded land use and land cover change dataset in China by harmonizing the annual provincial area of the biome from multi-source datasets, assimilating various gridded data for different time windows to build weighting maps of biomes, and then allocating the area of biomes spatially according to the weighting map to match the provincial records. Their new data revealed a strong carbon sink in China since 1980, the estimate of $F_{luc}$ during 2000-2019 is used in our study.

Leng et al. (under review) analyzed the net flux of $F_{luc}$ using a process-based model driven by high resolution satellite land cover maps for 2000s and 2010s, the resulthave been collectively included in our study.

For the other CO_2_ sectors (e.g. the inland water CO_2_ outgassing flux, the fire induced CO_2_ flux), we follow the synthesis result from the global datasets used in Wang et al.^1^. Since there isn’t dataset specific to China, we don’t go into details.

## 1.2 CH_4_ datasets

### 1.2.1 Top-down

We have included four estimates of inversion-based result to quantify China’s CH_4_ emissions during 2000-2019, only one of which assimilated measurements across China^18^.

Zhang et al.^18^ included newly available data from a surface network across China (7 CMA sites) to improve the ability to constrain emissions at subnational and sectoral levels. The ensemble average posterior estimate for total CH_4_ emissions from China is 54 Tg CH_4_ yr^-1^. Other 3 inversion results (MIROC4-ACTM, CAMS, and a regional decomposition from the Global Carbon Project) we have used refer to Wang et al.^1^.

### 1.2.2 Bottom-up

For CH_4_ sectors, In addition to global inventories such as the latest CEDS^19, 20^, EDGAR^21^, and FAOSTAT^22^ synthesized by Wang et al.^1^, we have also integrated 4 regional datasets and 18 model-based estimates for China.

NCCC compiled high complexity inventories in emissions from rice paddies, enteric fermentation from ruminants, and manure management, using national specific data. CH_4_ emissions from rice paddies were estimated from different farming practices, different irrigation management practices, and different fertilizer application practices. The emission factors were estimated using the Chinese rice field methane model (CH4MOD) with county-level statistical basic data of rice fields. For enteric fermentation sector, the activity data came from China Animal Industry Yearbook, China Statistical Yearbook, the National Agricultural Census and the Department of Animal Husbandry, Ministry of Agriculture (DAHMA). The breeding scale, the feeding ratio, and the proportion of different animals' age structure came from the livestock industry statistics provided by the DAHMA. The characteristics data (e.g. animal weight, daily gain of weight, feed intake and feed quality, milk yield and milk fat percentage, and wool yield) involved in the calculation of enteric fermentation CH_4_ emission factors were derived from the typical survey data of 79 counties conducted in 2015. For manure management sector, the proportion of manure management methods used to calculate emission factors was obtained from 79 counties. Based on the feed intake data obtained from typical surveys, the daily amount of volatile solids excreted by different livestock species at different ages in subregions of China were calculated.

Besides NCCC datasets, we have also collected a latest gridded estimate for the livestock emissions during 2000-2019. Zhang et al.^23^ adopted the default emission factors according to IPCC Tier 2 approach^24^ values with adjustment according to country specific production yield and feed quality, developed gridded CH_4_ emissions by allocating the provincial level CH_4_ emissions into the grid level according to spatial spatial proxy data extracted from Gridded Livestock of the World database (GLW).

For the aquaculture CH_4_ emissions, we acquired the estimate from two latest comprehensive studies, Zhang et al.^25^ made a stable nationwide meta-analysis based on 132 aquaculture sites in China^25^. Based on the mean fluxes of CH_4_ during the farming period for the different aquaculture system types, they multiplied them by the system-specific areal coverage in each province to calculate the spatial distributions and magnitudes of aquaculture-CH_4_ flux across China. They estimated that aquaculture systems in China emitted 2.9 ± 1.1 Tg CH_4_ yr^-1^.

Another field observations based estimate^26^ focusing on aquaculture ponds CH_4_ emissions during 2008-2019 in China is also integrated. With a database of 55 field observations, they examined that the annual CH_4_ fluxes from aquaculture ponds are much larger than those from reservoirs and lakes, which is 1.6 ± 0.6 Tg CH_4_ yr^-1^.

##### The WAD2M model estimates of wetland CH_4_ emission

In this study, we estimated the wetland CH_4_ emission based on GCP models^27^ during 2000-2017. 16 land surface models with different surface wetland extents (the diagnostics: prescribed surface wetland extent by WAD2M, and the prognostics: meteorological forcing) contributes to the ensemble estimate of wetland methane emissions.

For the other natural sectors, similar to the CO2 section, we extract the national estimate refer to global-scale inventories or models. The GFEDv4.1s dataset is used to estimate CH_4_ fire emissions during 2000-2019. The process-based terrestrial ecosystem model VISIT is ran from 2000 to 2016 to evaluate the natural soil CH_4_ sinks. The inland water CH_4_ emission is estimated as a stable term based on regionalized synthesis^1^ of 8 studies.

## 1.3 N_2_O datasets

### 1.3.1 Top-down

Inversion estimates of N_2_O fluxes are relatively limited in contrast to CO_2_ and CH_4_. Currently, there is no available inversion dataset with regional specific for China. We just estimate the N_2_O flux of China using the latest GCP^28^ inversions.

### 1.3.2 Bottom-up

For the land N_2_O budgets, apart from 4 widely used global inventories (CEDS, EDGAR, FAOSTAT and PRIMAP-HIST^29^), we have collected 5 national datasets in specific sectors and 6 model-based results for China.

NCCC reported N_2_O emissions from agricultural soils, manure management. The agricultural soils flux was estimated in 2 parts, the direct emissions from in situ conversion of nitrogen inputs, as well as indirect emissions from nitrogen deposition and leaching and runoff due to nitrogen inputs. The regional nitrogen cycle model (IAP-N model) was used to allocate nitrogen inputs and dynamics of each province. The direct emission factors was obtained by statistical analysis based on the observation data of different types of agricultural soils in China over the past 30 years. For the manure management sector, the nitrogen excretion data of livestock and poultry was based on the Manual of the National Pollution Source Survey of Production and Discharge of Pollutant Coefficient from Livestock.

For the N_2_O emissions from manure management, we additionally adopt a literature dataset from Xu et al.^30^. Using he NUtrient flows in Food chains, Environment and Resources use (NUFER) model, the principle of mass balance, and province-level activity data and province-specific EFs from previous studies, they estimated the national N_2_O emissions from manure management during 1978 to 2016.

For the N_2_O emissions from agricultural soils, a Tier 3 estimate^31^ using a linear mixed-effect model and survey-based data set of agricultural management measures to quantify the spatiotemporal changes of crop-specific cropland-N_2_O emissions from China between 1980 and 2017 is also included in our synthesis. They estimated the direct soil N_2_O emissions as the sum of emissions caused by anthropogenic fertilizer-induced emissions plus the remaining background emissions.

For the aquaculture N_2_O emissions, China has remained the largest aquaculture producer worldwide since 1990, Zhou et al.^32^ produced a species-, provincial-, and national-levels dataset in China using annual aquaculture production data, based on nitrogen (N) levels in feed type, feed amount, feed conversion ratio, and emission factor (EF).

Wang et al.^1^ have roughly quantified N_2_O emission from aquaculture based on the average stable N_2_O emission factor of aquaculture system globally and annual production data from FAOSTAT.

##### The NMIP model estimates of N_2_O fluxes

In this study, we have included different environmental factors emissions from six process-based models (DELM, ORCHIDEE, ORCHIDEECNP, VISIT, LPX-Bern and O-CN) in the N_2_O Model Intercomparison Project (NMIP)^33^, for the purpose of comparative analysis. Results from multiple experiments (from S0 to S6) are calculated, that are, the “best estimates” of N_2_O emissions (S1), the overall effect of all environmental factors (S1-S0), the effects of manure N use (S1-S2), and the effects of N fertilizer use (S2-S3).

For the other sectors, data are from the same sources as Wang et al.^1^.

# 2. Methods

## 2.1 Reconcile top-down and bottom-up estimates

### 2.1.1 CO_2_

The top-down atmospheric inversions estimate the net CO_2_ flux exchanged between the surface and the atmosphere. The signal from fossil fuel emissions in the space of concentrations should be removed at pre- or post-processing, while the emissions of reduced carbon compounds from land ecosystems (Fbrcc) and fossil fuel use (Ffrcc) are a large and overlooked component^34^, which will lead to a overestimate of the regional near surface CO_2_ concentration, and therefore cause overestimation of the inverted carbon sink. We eliminate the net flux of Fbrcc and Ffrcc based on an estimate^2^ using the Asian anthropogenic emission inventory for 2006, the Multi-resolution Emission Inventory for China (MEIC) for 2008 and 2010, and the GFED.

The inverted CO_2_ sink is also influenced by the lateral flux, CO_2_ emissions from the oxidization of net imported products. Carbon is lost or gained through the trade of crop, wood and animal products. The net imports of forest and crop products from outside China are included in the top-down sink estimates, but should be subtracted from it to be compared with the bottom-up estimate.

The top-down CO_2_ result is adjusted as follow:

$TD_{{CO}_{2}}=inversion {CO}_{2} flux+F_{frcc}+F_{brcc}+F_{trade}$ (1)

The bottom-up CO_2_ budget encompasses a set of component fluxes. From the inventory-based method, the total land CO_2_ budget contains the sum of recommended net carbon stock changes from terrestrial carbon pools include ${\Delta C}_{forest}$, ${\Delta C}_{grassland}$, ${\Delta C}_{cropland}$, ${\Delta C}_{other}$, ${\Delta C}_{burial}$, ${\Delta C}_{product}$, and the lateral river export of carbon ($F_{export}$), the reconciled bottom-up land CO_2_ budget follows the formula below:

${BU}_{{CO}_{2}}={\Delta C}_{forest}+{\Delta C}_{grassland}+{\Delta C}_{cropland}+{\Delta C}_{other}+{\Delta C}_{burial}+{\Delta C}_{product} +F_{export}$ (2)

### 2.1.2 CH_4_

While the inversion principles of CH_4_ and N_2_O are the same as for CO_2_, there are some special considerations for these species. Both of them estimate the net gas flux exchanged between the surface and the atmosphere. To estimate the land budget, the non-ecosystem fluxes need to been subtracted from the initial inversion CH_4_ and N_2_O flux. The non-ecosystem GHG fluxes includes CH_4_ and N_2_O emissions from fossil fuel and industry ($F_{fossil}$), and waste treatments and landfills ($F_{waste}$). The ensemble result from 7 latest datasets EDGAR, CEDS, PRIMAP, NCCC, REAS^35^, GAINS^36^, PKU^37^ (CH_4_ only) have been calculated for the non-ecosystem GHG fluxes.

For CH_4_ budget, the geological seepage induced CH_4_ emission ($F_{geology}$) should also be eliminated. We use the inventory estimate^38^ based on point sources and area sources from four main categories of natural geo-CH_4_ emissions. The top-down CH_4_ and N_2_O results are adjusted as follow:

${TD}_{{CH}_{4}}=inversion CH_{4} flux-F_{fossil}-F_{waste}-F_{geology}$ (3)

${TD}_{N_{2}O}=inversion N_{2}O flux-F_{fossil}-F_{waste}$ (5)

Terrestrial ecosystem CH_4_ budgets are calculated as the sum of the agricultural sectors (including emissions from enteric fermentation in ruminants, manure management, rice paddies and aquaculture), the wetland emissions, and other natural sectors (the sum of emissions related to biomass burning, inland water emissions, non-wetlands natural soil sink, and termite emissions). The bottom-up CH_4_ budget is as follow:

${BU}_{{CH}_{4}}=F_{enteric}+F_{manure}+F_{agrisoil}+F_{aqua}+F_{wetland}+F_{natusoil}+F_{fire}+F_{water}+F_{termite}$ (5)

### 2.1.3 N_2_O

Similar to the flux terms in land CH_4_ budget, we synthesize the land N_2_O budget following the terrestrial boundaries and terms definition of RECCAP-2. The land N_2_O budget consists of N_2_O emissions from the agricultural sectors such as manure management, upland soils and aquaculture, the natural sectors such as fire N_2_O emissions, inland waters N_2_O outgassing and N_2_O emissions from natural soils. The bottom-up N_2_O budget is as follow:

${BU}_{N_{2}O}=F_{manure}+F_{agrisoil}+F_{aqua}+F_{natusoil}+F_{fire}+F_{water}$ (6)

## 2.2 Integration of GHG budget

The overall GHG budget is evaluated using GWP100 (global warming potentials on 100-year time horizon) of three greenhouse gases for bottom-up and top-down approaches, respectively. GWP20 is also provided in Table S1. GWP indicates the integrated radiative forcing of CH_4_ and N_2_O in terms of a CO_2_ equivalent unit. This helps us measure the global warming impacts of different gases, which is adopted by assessment reports of IPCC. Specifically, it is a measure of how much energy the emissions of 1 ton of a gas will absorb over a given period of time, relative to the emissions of 1 ton of carbon dioxide (CO_2_).We adopt GWP100s of 27.0 and 273 for CH_4_ and N_2_O, respectively (GWP20s of 79.7 and 273 for CH_4_ and N_2_O), according to Table 7.15 in WG1 contribution to IPCC AR6^39^. The overall land GHG budget is calculated applying the following equation:

$GHG =Budget\left( {CO}_{2} \right)+Budget\left( {CH}_{4} \right)*GWP_{{CH}_{4}}+Budget\left( N_{2}O \right)*GWP_{N_{2}O}$ (7)

## 2.3 Uncertainty estimate

In this study, the fluxes uncertainty is calculated from the spread of different estimates, in those cases where the state of knowledge cannot establish that one estimate is better than another^34^. The standard deviation of different estimates over the past 20 years is calculated at the national scale, and it is used to quantify flux uncertainty. The uncertainty of the total budget is obtained by error propagation from uncertainties of each flux from equation (1) to equation (6). If different estimates report their own uncertainty, this information is used to evaluate consistency between estimates.

# Table S1. Terrestrial GHG budget based on GWP100 and GWP20 metrics.

| **Terrestrial GHG budget** (Tg CO_2_eq yr^-1^) | | | **CO_2_** | | **CH_4_** | | **N_2_O** | | **GHG total** | | **P1**^a^ | **P2**^b^ | |
| --- | --- | --- | --- | --- | --- | --- | --- | --- | --- | --- | --- | --- | --- |
|  | | **Mean** | | **sd** | **Mean** | **sd** | **Mean** | **sd** | **Mean** | **sd** |  |  |  |
| **GWP100** | |  | |  |  |  |  |  |  |  |  |  | |
| TD |  | -1151.0 | | 425.1 | 712.5 | 151.8 | 363.2 | 207.5 | -75.3 | 496.8 | 93% | | 768% |
| BU |  | -1229.2 | | 149.1 | 703.3 | 117.5 | 496.8 | 82.9 | -29.0 | 207.2 | 98% | | 719% |
| Natural | | -1155.0 | | 148.3 | 118.8 | 72.7 | 197.7 | 24.1 | -838.4 | 167.0 | 27% | |  |
| Agricultural | | -74.2 | | 15.3 | 584.5 | 92.3 | 299.1 | 79.3 | 809.4 | 122.7 | 1190% | |  |
| **GWP20** | |  | |  |  |  |  |  |  |  |  | |  |
| TD |  | -1151.0 | | 425.1 | 2103.3 | 448.2 | 363.2 | 207.5 | 1315.5 | 651.7 | 214% | | 768% |
| BU |  | -1229.2 | | 149.1 | 2076.2 | 346.9 | 496.8 | 82.9 | 1343.8 | 386.6 | 209% | | 719% |
| Natural | | -1155.0 | | 148.3 | 350.8 | 214.7 | 197.7 | 24.1 | -606.5 | 262.1 | 47% | |  |
| Agricultural | | -74.2 | | 15.3 | 1725.4 | 272.5 | 299.1 | 79.3 | 1950.3 | 284.2 | 2728% | |  |
| ^a^ Proportion of land CO_2_ sink being offset by terrestrial GHG source | | | | | | | | | | | | | |
| ^b^ Proportion of land CO_2_ sink being offset by total fossil fuel source | | | | | | | | | | | | | |

# Table S2. The GHG budget in China since 2000

| **Sectors** | | **CO_2_ (Tg CO_2_)** | | | **CH_4_ (Tg CH_4_)** | | | | **N_2_O (Tg N_2_O)** | | |
| --- | --- | --- | --- | --- | --- | --- | --- | --- | --- | --- | --- |
|  |  | **Mean** | | **Uncertainty** | **Mean** | | **Uncertainty** | | **Mean** | **Uncertainty** | |
| **1.**  **Human**  **Activities** | 1.A Fossil Fuel | | 8076.20 | 656.96 | | 23.10 | | 2.17 | 0.44 | | 0.14 |
|  | 1.B Waste and Landfill | |  |  | | 9.33 | | 3.37 | 0.26 | | 0.41 |
|  | **Subtotal** | | **8076.20** | **656.96** | | **32.42** | | **4.01** | **0.70** | | **0.43** |
| **2.**  **Carbon**  **Stock**  **Change** | 2.A Forests | | -659.40 | 125.40 | |  | |  |  | |  |
|  | 2.B Shrublands | | -113.85 | 51.15 | |  | |  |  | |  |
|  | 2.C Grasslands | | -36.28 | 45.23 | |  | |  |  | |  |
|  | 2.D Croplands | | -74.23 | 15.28 | |  | |  |  | |  |
|  | 2.E Wetlands | | -44.81 | 0.38 | |  | |  |  | |  |
|  | 2.F Urban Construction | | 2.08 | 0.64 | |  | |  |  | |  |
|  | 2.G Burial | | -73.33 | 36.67 | |  | |  |  | |  |
|  | 2.H Wood Products | | -103.21 | 10.39 | |  | |  |  | |  |
|  | **Subtotal** | | **-1103.03** | **148.57** | |  | |  |  | |  |
|  | NBP | | -863.90 | 337.10 | |  | |  |  | |  |
|  | Land Use Change Flux | | -282.92 | 280.66 | |  | |  |  | |  |
|  | **Subtotal** | | **-1146.82** | **438.64** | |  | |  |  | |  |
| **3.**  **Lateral Adjustments** | 3.A Net Trade | | -194.33 | 38.87 | |  | |  |  | |  |
|  | 3.B Lateral Transport to Ocean | | -126.16 | 12.82 | |  | |  |  | |  |
|  | 3.C Fossil Fuel RCC | | 322.67 | 18.33 | |  | |  |  | |  |
|  | 3.D Biogenic RCC | | 135.67 | 66.00 | |  | |  |  | |  |
| **4.**  **Agriculture** | 4.A Enteric Fermentation | |  |  | | 8.58 | | 1.32 |  | |  |
|  | 4.B Manure Management | |  |  | | 1.74 | | 0.92 | 0.27 | | 0.11 |
|  | 4.C Agricultural Soil | |  |  | | 8.40 | | 2.82 | 0.75 | | 0.26 |
|  | 4.D Aquaculture | |  |  | | 2.93 | | 1.07 | 0.07 | | 0.05 |
|  | **Subtotal** | |  |  | | **21.65** | | **3.42** | **1.10** | | **0.29** |
| **5.**  **Other**  **Sectors** | 5.A Wetlands | |  |  | | 2.55 | | 2.13 |  | |  |
|  | 5.B Natural Soil | |  |  | | -2.22 | | 0.21 | 0.69 | | 0.08 |
|  | 5.C Fires | | 68.32 | 1.66 | | 0.25 | | 0.05 | 0.01 | | 0.00 |
|  | 5.D Inland Waters | | 312.26 | 139.32 | | 3.50 | | 1.63 | 0.03 | | 0.03 |
|  | 5.E Termites | |  |  | | 0.32 | |  |  | |  |
|  | **Subtotal** | |  |  | | **4.40** | | **2.69** | **0.72** | | **0.09** |
|  | 5.H Geological Seepage^b^ | |  |  | | 1.34 | | 0.27 |  | |  |
|  | TD Inversion | | -1414.98 | 417.70 | | 52.39 | | 5.50 | 2.03 | | 0.62 |
| **Balance** | **BU Land Budget** | | **-1229.18** | **149.12** | | **26.05** | | **4.35** | **1.82** | | **0.30** |
|  | **TD Land Budget** | | **-1150.98** | **425.06** | | **26.39** | | **5.62** | **1.33** | | **0.76** |
| ^a^ The uncertainty for 2.E is estimated as 21% of the mean value^7^.  ^b^ Geological seepage is not contained within the boundaries of our land ecosystems framework. | | | | | | | | | | | |

# *Supporting References*

1. Wang X *et al.* The greenhouse gas budget of terrestrial ecosystems in East Asia since 2000. *ESS Open Archive* 2023.

2. Jiang F *et al.* A comprehensive estimate of recent carbon sinks in China using both top-down and bottom-up approaches. *Scientific Reports* 2016; **6**: 22130.

3. Chen B *et al.* An atmospheric perspective on the carbon budgets of terrestrial ecosystems in China: progress and challenges. *Science Bulletin* 2021; **66**: 1713-18.

4. Wang Y *et al.* The size of the land carbon sink in China. *Nature* 2022; **603**: E7-E9.

5. Luo Y *et al.* Predictability of the terrestrial carbon cycle. *Glob Chang Biol* 2015; **21**: 1737-51.

6. Sitch S *et al.* Recent trends and drivers of regional sources and sinks of carbon dioxide. *Biogeosciences* 2015; **12**: 653-79.

7. China. National communication (NC). NC 3. 2018.

8. China. Biennial update report (BUR). BUR 2. 2018.

9. Piao S *et al.* The carbon balance of terrestrial ecosystems in China. *Nature* 2009; **458**: 1009-13.

10. Pan Y *et al.* A Large and Persistent Carbon Sink in the World's Forests. *Science* 2011; **333**: 988-93.

11. Mendonça R *et al.* Organic carbon burial in global lakes and reservoirs. *Nature Communications* 2017; **8**.

12. Fang J *et al.* Climate change, human impacts, and carbon sequestration in China. *Proceedings of the National Academy of Sciences* 2018; **115**: 4015-20.

13. Xu L *et al.* Changes in global terrestrial live biomass over the 21st century. *Science Advances* 2021; **7**: eabe9829.

14. Chang Z *et al.* Estimating Aboveground Carbon Dynamic of China Using Optical and Microwave Remote-Sensing Datasets from 2013 to 2019. *Journal of Remote Sensing* 2023; **3**: 0005.

15. Fang J *et al.* Biomass and net production of forest vegetation in China. . *Acta Ecologica Sinica* 1996; **16**: 497-508.

16. Yan Y *et al.* Increasing riverine export of dissolved organic carbon from China. *Global Change Biology* 2023; **n/a**.

17. Yu Z *et al.* Forest expansion dominates China’s land carbon sink since 1980. *Nature Communications* 2022; **13**.

18. Zhang Y *et al.* Observed changes in China's methane emissions linked to policy drivers. *Proceedings of the National Academy of Sciences* 2022; **119**: e2202742119.

19. Hoesly RM *et al.* Historical (1750–2014) anthropogenic emissions of reactive gases and aerosols from the Community Emissions Data System (CEDS). *Geoscientific Model Development* 2018.

20. McDuffie EE *et al.* A global anthropogenic emission inventory of atmospheric pollutants from sector- and fuel-specific sources (1970–2017): an application of the Community Emissions Data System (CEDS). *Earth System Science Data* 2020; **12**: 3413-42.

21. Crippa M *et al.* CO2 emissions of all world countries. *JRC/IEA/PBL 2022 Report, EUR 31182 EN, Publications Office of the European Union, Luxembourg* 2022.

22. FAO. Emissions from agriculture and forest land. Global, regional and country trends 1990–2019. *FAOSTAT Analytical Brief Series* 2021; **25**.

23. Zhang L *et al.* Methane emissions from livestock in East Asia during 1961-2019. *Ecosystem Health and Sustainability* 2021; **7**.

24. *2019 Refinement to the 2006 IPCC Guidelines for National Greenhouse Gas Inventories. Volume 4 Agriculture, Forestry and Other Land Use*: Intergovernmental Panel on Climate Change, Geneva, Switzerland, 2019.

25. Zhang Y *et al.* Assessing carbon greenhouse gas emissions from aquaculture in China based on aquaculture system types, species, environmental conditions and management practices. *Agriculture, Ecosystems & Environment* 2022; **338**.

26. Dong B *et al.* Quantifying Methane Emissions from Aquaculture Ponds in China. *Environmental Science & Technology* 2023; **57**: 1576-83.

27. Saunois M *et al.* The Global Methane Budget 2000–2017. *Earth System Science Data* 2020; **12**: 1561-623.

28. Tian H *et al.* A comprehensive quantification of global nitrous oxide sources and sinks. *Nature* 2020; **586**: 248-56.

29. Gütschow J *et al.* The PRIMAP-hist national historical emissions time series. *Earth System Science Data* 2016; **8**: 571-603.

30. Xu P *et al.* Policy-enabled stabilization of nitrous oxide emissions from livestock production in China over 1978–2017. *Nature Food* 2022; **3**: 356-66.

31. Cui X *et al.* Deceleration of Cropland-N2O Emissions in China and Future Mitigation Potentials. *Environmental Science & Technology* 2022; **56**: 4665-75.

32. Zhou Y *et al.* Four decades of nitrous oxide emission from Chinese aquaculture underscores the urgency and opportunity for climate change mitigation. *Environmental Research Letters* 2021; **16**.

33. Tian H *et al.* The Global N2O Model Intercomparison Project. *Bulletin of the American Meteorological Society* 2018; **99**: 1231-51.

34. Ciais P *et al.* Definitions and methods to estimate regional land carbon fluxes for the second phase of the REgional Carbon Cycle Assessment and Processes Project (RECCAP-2). *Geoscientific Model Development* 2022.

35. Kurokawa J *et al.* Emissions of air pollutants and greenhouse gases over Asian regions during 2000–2008: Regional Emission inventory in ASia (REAS) version 2. *Atmospheric Chemistry and Physics* 2013; **13**: 11019-58.

36. Höglund-Isaksson L. Global anthropogenic methane emissions 2005–2030: technical mitigation potentials and costs. *Atmospheric Chemistry and Physics* 2012; **12**: 9079-96.

37. Peng S *et al.* Inventory of anthropogenic methane emissions in mainland China from 1980 to 2010. *Atmospheric Chemistry and Physics* 2016; **16**: 14545-62.

38. Etiope G *et al.* Gridded maps of geological methane emissions and their isotopic signature. *Earth System Science Data* 2019; **11**: 1-22.

39. Canadell JG *et al.* Global carbon and other biogeochemical cycles and feedbacks. IPCC AR6 WGI, Final Government Distribution, chapter 5. 2021.
